# Dentine deproteinization and microleakage around gingival third resin restorations

**DOI:** 10.4103/0972-0707.43412

**Published:** 2008

**Authors:** Sowmya Shetty, Mithra B, Sureshchandra B

**Affiliations:** 1 Department of Conservative Dentistry and Endodontics, Former PG student, A. B. Shetty Memorial Institute for Dental Sciences, Mangalore, India; 2 Department of Conservative Dentistry and Endodontics, HOD, A. B. Shetty Memorial Institute for Dental Sciences, Mangalore, India; 3 Department of Conservative Dentistry and Endodontics, Former HOD, A. B. Shetty Memorial Institute for Dental Sciences, Mangalore, India

**Keywords:** Bonding, collagen removal, dentin deproteinization, gingival third restorations; microleakage, quantitative dye leakage, spectrophotometry

## Abstract

**Objectives:**

A significant factor in achieving satisfactory adhesion of restorative resins to dentine substrate is the method by which the dentine surface is treated before an adhesive is applied. The aim of this study was to evaluate the effect of deproteinization on microleakage around gingival third resin restorations.

**Materials and Methods:**

Standardised Class V preparations were made on randomly selected intact upper and lower human molars. These were treated in one of five ways (no treatment, enamel etch only, total etch, total etch followed by deproteinization, and deproteinization only) and then adhesively bonded using either an acetone or ethanol based bonding system. The samples were first immersed in 2% methylene blue dye and then 35% nitric acid, for 72 hours each. The solutions were filtered and centrifuged, and the supernatant was used to determine absorbance in a spectrophotometer at 670 nm. The results were recorded as a measure of transmission of light of the test solutions.

**Results:**

The results were subjected to multiple comparisons amongst groups, using anova. There was a statistically significant difference between all treatment groups for the two different bonding systems used. The experimental groups, total etch alone and total etch followed by deproteinization showed statistically significant differences, as compared to all other groups. However, although the total etch group showed a decrease in microleakage, when compared to the total etch followed by deproteinization group, this was not statistically significant.

**Conclusion:**

Within the limitations of this study, collagen removal may be important to reduce microleakage whilst using acetone based adhesive systems and it may not influence the amount of microleakage for ethanol or water based adhesive systems.

## INTRODUCTION

One of the challenges of restorative dentistry research is to develop adhesive restorative materials that provide an effective bond to dental tissues and consequently offer successful restorative treatment. Whilst it is known that infiltration of a resin monomer into chemically conditioned dentine is considered essential for improving bonding at the resin-tooth interface, it is also accepted that the newer one-bottle adhesives, dissolved in acetone, alcohol or water solvents, diffuse only into the outer few micron meters of the tissue that has been rendered porous by acid conditioning. While enamel is a reliable substrate for bonding, dentine bonding remains less predictable and bonding to cementum even less so.

With increasing population age and incidences of root caries, it is of great concern that we do not have a predictable bond at the cementum-dentine interface. Bonding to the gingival third of the tooth involves treatment and conditioning of three different dental tissues. If a good chemical bond can be achieved, the excellence of the union between the dental tissues and the restoration might preclude the chances of secondary caries encouraged by microleakage.

One of the variables that may affect the longevity of Class V resin restorations is the quality of the bond at the dentinal cavosurface margin. There is a zone just beneath the cementum, which has few dentinal tubules and which seems to have a low permeability to adhesive resins, even after acid etching. Acidic conditioners may demineralise more of the dentine than subsequently applied resin monomers can infiltrate, producing a poor quality hybrid layer at this critical interface.[1] The external margins of Class V resin composites in dentine should be as perfect as possible, both macroscopically and microscopically, since these regions are often sub gingival.

Removal of the collagen fibres with a deproteinizing agent would facilitate the access of the adhesive resins to a substrate that is more permeable and less sensitive to water content. Using deproteinization processes to remove the superficial destabilized collagen layer and subsurface remnants from etched dentine surfaces has been proposed since the 1990's.[2]

Sodium hypochlorite is a non-specific proteolytic agent that effectively removes organic components at room temperature. Literature on the subject shows that sodium hypochlorite treatment removes the dentine's organic components and changes its chemical composition, so that it becomes similar to etched enamel. This substrate is also rich in exposed hydroxyapatite crystals and may result in a stable interface over time, because it is made of mineral. An increase in “wettability” is expected on deproteinized dentine surfaces, since they are hydrophilic,[3] and are more permeable.[4] Thus, chemical interactions between resin and the deproteinized dentine surface are most likely to occur, since the surface has been described as having wider tubule openings with finer irregularities on the intertubular dentine, after just a two minute treatment with a deproteinizing agent.[2]

It has been observed that etching followed by deproteinization resulted in lower degree of leakage at occlusal enamel margins than at gingival dentine and cementum margins,[3] especially when acetone based bonding agents were used.[5] However, we wanted to investigate the effect of deproteinization on microleakage in gingival third restorations.

## MATERIALS AND METHODS

**Figure 1:**
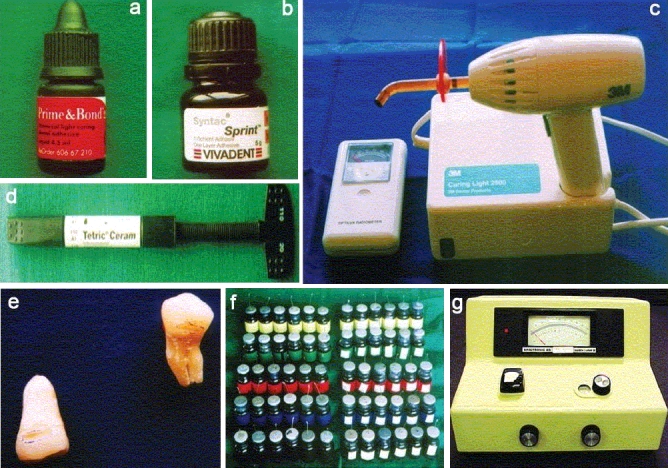
Materials and instruments (a) Acetone based adhesive system (Prime and bond 2.1 ®(Dentsply, US) (b) Ethanol based adhesive system (Syntac Sprint ®, Ivoclar Vivadent AG, Leichenstein) (c) Radiometer (Optilux, Kerr corp., UK) and Light curing unit (3M Dental Products, MN, USA) (d) Composite resin (Tetric Ceram, Ivoclar Vivadent AG, Leichenstein) (e) Prepared tooth samples (f) Restored tooth samples in 2 % methylene dye (g) Spectrophotometer(Spectronic 20, Bausch and Lomb, NY, USA)

Sixty freshly extracted intact human molars were collected, stored, disinfected and handled as per the recommendations and guidelines laid down by OSHA and CDC.[6] Carious and cracked teeth were discarded and the rest of the teeth were stored in 0.9% physiologic saline solution, prior to cavity preparation. Uniform Class V preparations on the buccal surfaces of the teeth were prepared with an air rotor hand piece. The measurements of the cavities were approximately 5 mm mesio-distally, 3 mm cervico-occlusally and 2 mm deep [Figure 1e].

The teeth were randomly divided into five groups of 12 teeth each. The experimental groups included positive and negative controls: enamel etch and no treatment at all. These groups were treated in one of the following ways: etched completely with 37% phosphoric acid; etched and followed by deproteinization with 2% sodium hypochlorite with agitation for 2 min, or deproteinized only. Following this, the preparations were bonded with a bonding agent containing an acetone based primer (Prime and bond 2.1 - ®) or an ethanol based primer (Syntac sprint - ®), along with a light cure composite resin, depending on the subgroup. The materials used are shown in Figures 1a - 1d.

The entire surfaces of the restored teeth were covered with nail varnish, except for the restoration and a 2 mm margin around it. Each specimen was individually immersed in 5 ml of 2% methylene blue solution [Figure 1f] and stored at 37°C ± 2°C, at relative humidity for 72 hours. Following this, the specimens were washed with water, ultrasonicated and the nail varnish removed. The teeth were then suspended in freshly prepared 35% nitric acid for 72 hours. The solutions were filtered and centrifuged for one minute, at 2000 rpm, and the supernatant solutions were used to determine absorbance in a spectrophotometer [Figure 1g] at 670 nm.

The results were recorded as a measure of transmission of light as well as optical density of the test solutions. When the t-test was applied to the results, they proved to be statistically significant.

## RESULTS

The results are presented in Table 1 as volumetric dye penetration in terms of percentage transmission of light through the test solutions. Tables 2-4 present the means, standard deviations and *P* values as well as multiple comparisons and ANOVA results.

**Table 1:** Volumetric dye penetration values obtained in various groups, with respect to transmission of light

Groups	Subgroups	(%) Transmission of light
Group I	A	23,17,21,28,18,17
	B	18,23,12,23,14,23

Group II	A	34,29,30,31,35,30
	B	17,19,21,23,24,21

Group III	A	65,50,54,62,63,60
	B	49,48,54,40,54,60

Group IV	A	60,62,59,54,40,45
	B	30,29,34,54,60,30

Group V	A	28,18,22,15,25,17
	B	21,21,14,15,15,20

**Table 2:** Mean, standard deviation and *P* values of volumetric dye penetration in the various groups with reference to transmission

Groups	Sub groups	Mean	Standard deviation	T	*P*	Comments
Group I	A	20.6	4.3	.6830	.510	NS
	B	18.8	4.9			

Group II	A	31.5	2.4	7.4000	.000	VHS
	B	20.8	2.5			

Group III	A	59.0	5.7	2.2340	.049	SIG
	B	50.8	6.8			

Group IV	A	53.33	8.9	2.0620	.066	NS
	B	39.5	13.7			

Group V	A	20.8	5.0	1.28	.228	NS
	B	17.6	3.3	50			

**Table 3:** ANOVA with respect to the two subgroups

Sub group	F	Significance	Comment
A	60.089	.000	VHS
B	23.758	.000	VHS

Table 4Multiple comparisons amongst various groups

**Table:** Sub Group A – Acetone based system

(I) Group	(J) Group	Mean difference (I-J)	*P*	Comments
I	II	-10.8333	.003	HS
	III	-38.333	.000	VHS
	IV	-32.666	.000	VHS
	V	-0.1667	.960	NS

II	III	-27.500	.000	VHS
	IV	-21.833	.000	VHS
	V	10.666	.003	HS

III	IV	5.666	.098	NS
	V	38.166	.000	VHS

IV	V	32.500	.000	VHS

**Table:** Sub Group B – Ethanol based system

(I) Group	(J) Group	Mean difference (I-J)	*P*	Comments
I	II	-2.000	.647	NS
	III	-32.000	.000	VHS
	IV	-20.666	.000	VHS
	V	1.166	.789	NS

II	III	-5.43	.000	VHS
	IV	-18.667	.000	VHS
	V	3.166	.470	NS

III	IV	11.333	.014	SIG
	V	33.166	.000	VHS

IV	V	21.833	.000	VHS

VHS - Very highly significant ; HS - Highly significant SIG - Significant; NS - Not significant

**Figure 2:**
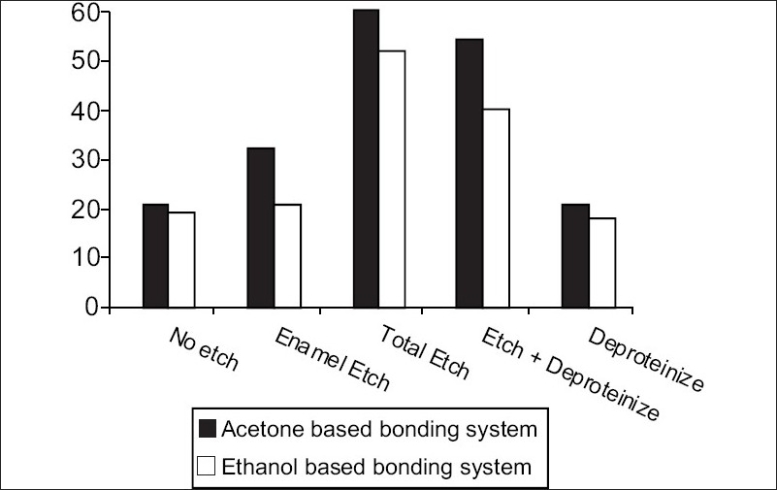
Chart showing comparison of percentage transmission of light - inversely propotional to mean volumetric dye leakage

[Figure 2] shows comparison of mean volumetric dye leakage in various groups, with respect to percentage transmission of light, microleakage being inversely related to percentage transmission of light.

It is interesting to note that there is a significant difference between the two adhesive systems within each treatment group. In comparison to the negative control, all groups showed highly significant statistical differences, irrespective of the adhesive system used, which was expected. With the acetone based system, there was a statistically significant difference between the experimental groups, with least microleakage being shown by those that were subjected to a total etch surface treatment rather than deproteinization treatment following etching. However for the ethanol based adhesive systems, there was no significant difference between the two groups. The groups that were subjected to etching of the enamel margins alone showed significant differences with respect to all other groups, and almost equalled that of the groups that were deproteinized only.

Another interesting point to note is that the groups that involved enamel etching alone, as opposed to total etching, showed higher microleakage.

It was seen that the least microleakage was seen in groups that were subjected to total etch alone, followed by deproteinization. These did not show significant differences between each other and further research may be necessary to determine if dentine deproteinization is a clinically relevant and viable means of improving the quality of the bond between the resin and the dentine, especially at gingival third areas.

## DISCUSSION

Dentine has been characterized as a biologic composite of a collagen rich matrix filled with nanomer sized calcium deficient, carbonate rich apatite crystals dispersed between paralleled micron sized, and hyper mineralized collagen poor hollow cylinders. The goal of hybridizing demineralised dentine with adhesive resins is to provide a structure that resists chemical attack and provides stable adhesion of overlying resin components and is itself stable and impermeable to oral fluids or bacterial substances. The concept of the “hybrid layer” was proposed in 1982 by Nakabayashi *et al.*[7] Their work suggested that the diffusion of monomers, infiltrated into an etched dentine surface and polymerized in situ, caused good adhesion with the tooth substrate.

In gingival third restorations, much has been said about the quality of the bond that occurs between the dentine and the resin. What is important, however, is finding the means to improve bonding at the cementum interface. A less mineralised structure might cause a compromised “hybrid layer”, with the involvement of collagen fibrils. Various treatment protocols have been proposed to increase “wettability”, permeability and promotion of a true chemical bond at this interface. In the present study, it was generally found that samples that were acid etched and deproteinized demonstrated higher microleakage, as compared to those that were only acid etched but not deproteinized. This is in agreement with previous studies conducted under similar parameters. The results also indicated that the microleakage values also depended on the specific adhesive that followed the dentine pretreatment procedures.

We could not demonstrate a statistically significant difference for the microleakage of the acetone based adhesive system, between the positive control and the experimental group. This might be an effect of the diffusibility of the acetone, as well as its ability to displace water.[8] Improved contact of the monomer, with irregular intertubular dentine structure, exposed by sodium hypochlorite treatment, might have resulted in a homogenous interface with no voids and with no need for a deproteinized hydrophilic surface. No collagen fibrils are directly exposed to the oral environment; hence the expected degradation of adhesive interface by hydrolysis of exposed collagen should not occur.[9]

In the present study, the negative control group (surface treatment was purely deproteinization of dentine) showed the maximum leakage for both bonding systems; however, the ethanol based system showed more quantitative leakage of the dye.

## CONCLUSIONS

It can be concluded that collagen removal may be important to reduce the microleakage, whilst using the acetone based adhesive, but it has no influence on the ethanol based system. However, further investigations would help to confirm the results in order to evaluate the effectiveness of this dentine treatment.
